# Multiple injuries in vital organs in a child from a gunshot: a case report

**DOI:** 10.4076/1757-1626-2-6340

**Published:** 2009-06-23

**Authors:** Nikolaos Katzilakis, Michalis Michalakis, John Vlachakis, John Arbiros, Efrosyni Vassilaki, John Germanakis, Emmanuel Velivassakis, Nikolaos Rikos, Anna Maria Spanaki, George Briassoulis

**Affiliations:** Department of Pediatric Intensive Care Unit, University Hospital of HeraklionHeraklion, CreteGreece

## Abstract

**Introduction:**

Many people in rural and urban areas own a gun legally or illegally. It is a social phenomenon that apart from the adults, the children become familiar with the guns in early age.

**Case presentation:**

A nine year old boy was shot by accident by his uncle, who was cleaning his gun (carbine) close to where the child was playing. More than 200 pellets were counted in the boy’s x-rays. The boy was hospitalised in pediatric intensive care unit with many injuries in thorax, abdomen, and limbs, clearly shown by the x-rays. He developed multiple injuries in lungs and liver, bilateral haemothorax and pneumothorax, subcutaneous emphysema, injury of pericardium, perirenal hematoma, choloperitoneum, injuries in the intestine and in the limbs. Initial level of lead in the boy’s body was measured as a reference value. Thereafter, frequent monitoring for lead levels was scheduled to prevent a potential lead poisoning from the pellets’ absorption. After the appropriate treatment the boy left the hospital in a month in good health.

**Conclusion:**

The consequences from gun use in places where children are exposed could be fatal. The appropriate co-operation of different medical sub-specialities in Pediatrics and the presence of pediatric intensive care unit can save the life of a child with multiple injuries.

## Introduction

Penetrating injuries of the thorax and abdomen, which occur primarily from gunshot and stabbing in children are rare [1,2,4]. In some areas, children and adults are unfortunately familiar with the guns. This social phenomenon can cause accidents in many cases. Reports from the international literature show that in most cases a multi-organ injury is present [3]. We present the case of a child of a firearm injury and the management of the serious injuries he developed. He was injured by a relative of him by accident, while the relative was cleaning his carbine.

## Case presentation

**Figure 1.**
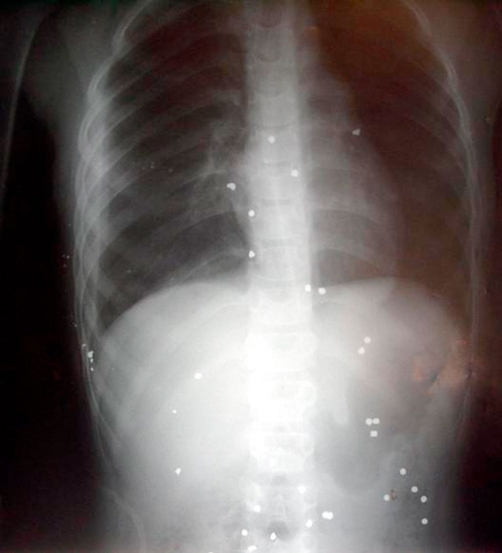
X-ray shows the pellets in the thorax and abdomen.

A 9-year-old Greek boy was transferred in the Emergency Department of the hospital after sustaining injuries by stray lead shots fired from a shotgun. On his admission the patient had a patent airway, Beats: 122 per minute, Blood Pressure: 135/60 mmHg, SpO2: 96% and Breaths: 30/min while from the full blood count Hb: 6.6 gm/dl, Hct: 17.2%. He had suffered multiple entry points by the lead shots that were distributed across the right side of his thorax, abdomen and left arm. On auscultation the patient had decreased lung sounds on the right side and subcutaneous emphysema. A chest tube was placed that confirmed the suspicion of pneumothorax and hemothorax. His abdomen was soft and tender with no sign of peritonitis engorgement or pain. A urine catheter was placed that revealed macroscopic hematuria. The patient was intubated and a computed tomography scan was followed. The computed tomography scan of the thorax revealed lacerations of the lung parenchyma in the right middle and lower lobe, pneumothorax, free pleural fluid, large subcutaneous emphysema, small collection of fluid (blood) in the pericardium and multiple lead shots scattered inside the thoracic cavity and the thoracic wall. No major injury to the heart (one lead shot was lodged between the epicardium and pericardium) or the large blood vessels was observed (Figure 1).

In the abdominal cavity the computed tomography scan showed: extended lacerations of the liver (segments V, VI, VII, VIII,) and a fracture of segment V fracture of the lower pole of the right kidney. The child was transferred in the pediatric intensive care unit of our hospital were he was transfused with blood products and remained hemodynamically stable during the critical initial period after the injury. The continuous monitoring by clinical examination and ultrasound of his abdominal cavity showed no change and his clinical condition was stabilized in the pediatric intensive care unit with no further blood loss. He continued to have a large quantity of fluid in his abdomen that showed no sign of decreasing, on the contrary on the 8^th^ day of his hospitalization the child was transferred in the operating room for an exploratory surgery of the abdomen due to the progression of intra-abdominal inflammation caused by choloperitoneum and the very large amount of fluid collected. During the operation the peritoneal cavity was found to be filled with bile and on exploration of the billiary tree a small hole was found on the wall of the gallbladder. His post-op hospitalization was normal with no complications. The manufacturers of the lead pellets for shotguns use lead and antimony as propellants. Because of the large amount of lead pellets inside the patient’s body the blood lead levels were measured periodically [5]. He also developed a temporary ulnar nerve paresis which was developed by a pellet which injured the nerve.

## Discussion

The most commonly affected organs by penetrating firearm injury in children are the small bowel, colon, liver and stomach followed by the spleen, kidney and pancreas [3,6,7]. Anatomic differences between the body of children and adults may account for the higher incidence of multivisceral injuries in children with penetrating injury [6]. The thoracic cage in children is much more compliant and not as well developed therefore affording less protection to the liver and other organs. The viscera of children are smaller and in closer proximity to each other, and the hepatic mass is smaller providing less physical protection to adjacent organs than in adults. The injuries that are developed by the pellets can be fatal in some cases. The incidence of gunshots in children and adults is rising over time [1,8]. The existence of a pediatric intensive care unit is very crucial for the management of these patients. The appropriate co-operation of different medical specialities in such multi-injured patients can save the life of a child.

## Conclusion

We feel that carefully selected patients can be treated conservatively under pediatric intensive care unit monitoring conditions having in mind that especially in gunshots the trauma to the visceral organs is often multiple and the management can be altered according to the needs of the patient.

## List of abbreviations

None
